# Orthogonally polarized dual-wavelength Nd:LaMgAl_11_O_19_/Nd:SrAl_12_O_19_ laser at 1297 and 1306 nm with adjustable ratio of output powers

**DOI:** 10.1371/journal.pone.0331770

**Published:** 2025-09-04

**Authors:** Chu Chu, Xinghua Yang, Yuzhao Li

**Affiliations:** 1 School of Physics and Opto-Electronic Engineering, Harbin Engineering University, Harbin, China; 2 School of Physics and Astronomy, Yunnan University, Kunming, China; Rutgers University Newark, UNITED STATES OF AMERICA

## Abstract

Using tunable in-band laser diode (LD) pumping (791.1–798.2 nm), an orthogonally polarized dual-wavelength (OPDW) Nd:LaMgAl_11_O_19_/Nd:SrAl_12_O_19_ (Nd:LMA/Nd:SA) operation at 1297 nm and 1306 nm for the ^4^F_3/2_ → ^4^I_13/2_ transition is demonstrated for the first time. Optimization of LD temperature and pump focus position enabled efficient generation of both wavelengths at balanced power. The maximum combined continuous-wave (CW) output reached 3.32 W at 1297 nm and 1306 nm with a near 1:1 power ratio. Relative to the absorbed 796.56 nm pump power, the laser achieved maximum total slope efficiency and optical-to-optical conversion efficiency of 20.7% and 18.5%, respectively. This 1297 nm and 1306 nm OPDW source shows significant potential for medical and scientific applications.

## 1.Introduction

**Fig 1:**
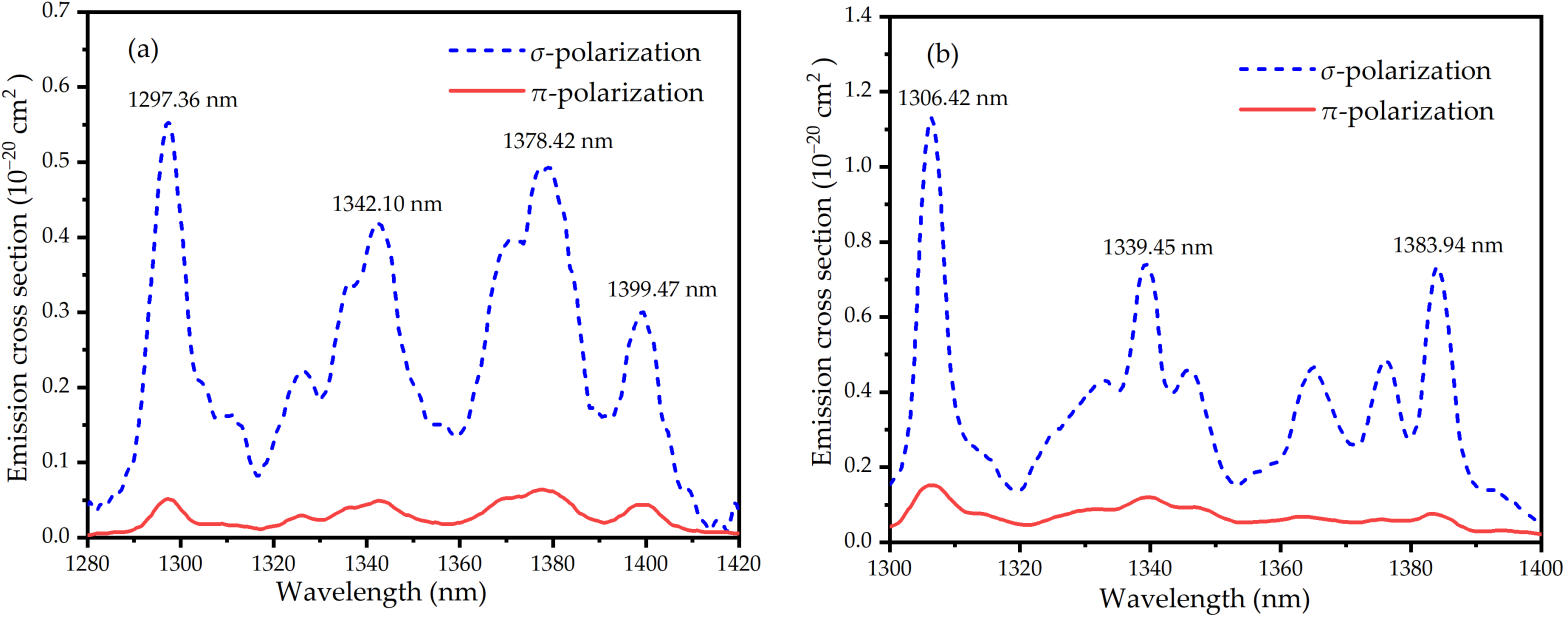
Emission spectra for the (a) Nd:LMA and (b) Nd:SA on the ^4^F_3/2_ → ^4^I_13/2_ transition.

CW OPDW lasers find diverse applications including precision fields (metrology [1], spectroscopy [2], measurement [3]), radar [4], medical diagnostics [5], holography [6,7], and nonlinear frequency conversion enabling visible-to-ultraviolet light generation [8–11] and terahertz-wave generation [12,13]. In particular, OPDW lasers operating near 1.3 μm [14–17] have attracted significant attention. This interest stems from their applications in atomic clocks, silver atom sub-Doppler cooling, and laser therapy [18–20]. So far, Nd-doped laser emissions were only obtained in about 1310–1360 nm range on ^4^F_3/2_ → ^4^I_13/2_ transition [21–32], but never below. However, laser emissions within the 1290–1310 nm range hold significant application potential in scientific and medical fields. For example, tooth enamel is transparent in the near-infrared 1290–1310 nm range, so it is possible to take images of parts of the tooth with different thicknesses through this band and find the location of lesions [33]. Again, Raman conversion of 1290–1310 nm OPDW lasers to the 1.5 µm range enables critical applications, including eye-safe differential lidar [34] and long-range optical communication [35]. In addition, the 1290–1310 nm spectral region resides within the O-band where lasers exploit near-zero chromatic dispersion in standard single-mode fiber to enable cost-effective medium-reach optical links for access networks, legacy systems, and short-reach data center interconnects [36–38]. To obtain dual-wavelength lasing, gain and loss between wavelengths must be balanced. This is typically achieved using intracavity loss components such as specially coated cavity mirror [39–44], etalon [45–47] and birefringent filter [48–50]. A persistent challenge, however, has been the limited ability to actively control the output power ratio of the two lines. Implementing a composite gain medium offers an effective solution to this problem. Currently, three principal methods exist for adjusting the power ratio: (1) Varying the axial location of the pump focus within the composite gain medium [51]. (2) Utilizing a bonded crystal and shifting the pump focus laterally [52]. (3) Modifying the wavelength of the pump beam to alter the relative pump absorption within the bonded crystal [53]. These methods have been successfully applied to achieve dual-wavelength operation in composite media [50–53]. Nd:LMA/Nd:SA are particularly suitable gain materials for solid-state lasers due to their moderate emission cross-sections, high luminescent efficiency, and broad linewidths. Consequently, laser emission in the 0.9–1.05 μm range has successfully been generated in Nd:LMA/Nd:SA on the ^4^F_3/2_ → ^4^I_9/2_ [54] and ^4^F_3/2_ → ^4^I_11/2_ [55–61] transitions. To our knowledge, Nd:LMA/Nd:SA lasing via on the ^4^F_3/2_ → ^4^I_13/2_ transition remains unreported. The stimulated emission cross-sections for the Nd:LMA and Nd:SA on the ^4^F_3/2_ → ^4^I_13/2_ transition, calculated via the Füchtbauer-Ladenburg equation [62], appear in Fig 1. Both crystals exhibit significantly stronger σ-polarized emission than π-polarization, with peak σ-emission at 1297 nm (Nd:LMA) and 1306 nm (Nd:SA). Notably, while most Nd-doped crystals exhibit their primary emission peaks between 1310–1360 nm for the ^4^F_3/2_ → ^4^I_13/2_ transition, the Nd:LMA and Nd:SA display exceptional fluorescence characteristics with their strongest transitions concentrated in the 1290–1310 nm spectral band. This distinctive spectral property makes Nd:LMA and Nd:SA particularly suitable as laser gain media for developing compact and high-efficiency laser systems operating in this specific wavelength range.

In this work, an OPDW Nd:LMA/Nd:SA operation at 1297 nm and 1306 nm on the ^4^F_3/2_ → ^4^I_13/2_ transition was realized. The OPDW power output ratio can be controlled by adjusting the LD working temperature or pump waist location. Combined output at 1297 nm and 1306 nm reached 3.32 W, exhibiting a slope efficiency of 20.7% and an optical conversion efficiency of 18.5% relative to absorbed 796.56 nm pump power.

## 2. Experimental setup

**Fig 2:**
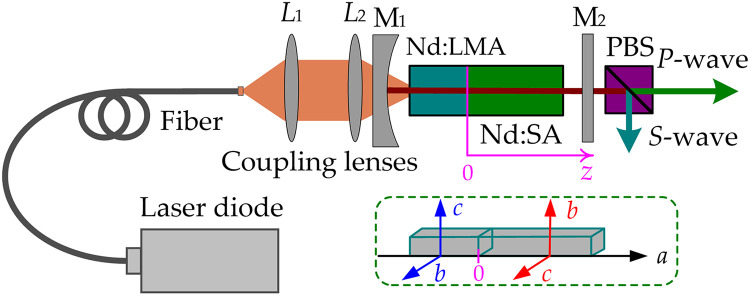
Schematic of the OPDW laser experiment. Inset: Nd:LMA/Nd:SA arrangement.

Fig 2 depicts the schematic for the OPDW Nd:LMA/Nd:SA operation at 1297 nm and 1306 nm. A fiber-coupled laser diode (G2 series, Cutting Edge Optronics) with a 400 μm core diameter, numerical aperture (NA) of 0.22, and beam quality factor (M^2^) ≈ 45 was used as the pump source. The wavelength was tunable from 791.1 to 798.2 nm, delivering a maximum output power of 18.7 W. LD temperature was stabilized between 16–38°C using a thermoelectric cooler with thermistor monitoring. Pump beam coupling employed lenses L_1_ and L_2_ (identical focal length *f* = 50 mm, antireflection (AR)-coated 790–800 nm). The gain medium comprised an *a*-cut Nd:LMA (6 mm length, 1.0 at.% Nd^3^⁺) and an *a*-cut Nd:SA (10 mm length, 2.0 at.% Nd^3^⁺). Their mutually perpendicular *c*-axes enabled orthogonal σ-polarized outputs: 1297 nm from Nd:LMA and 1306 nm from Nd:SA. A polarizing beam splitter (PBS) separated these emissions (S-wave: Nd:LMA, P-wave: Nd:SA). Both crystals, wrapped in indium foil, were mounted on water-cooled copper blocks at 16°C. The laser cavity featured a concave input coupler M_1_ (*R*_*OC*_ = −300 mm) with AR coatings (790–950 nm, 1030–1100 nm) and HR coating (1295–1310 nm). The plane output coupler M_2_ (3.5% transmittance at 1295–1310 nm, AR at 1030–1100 nm) yielded optimal performance among tested transmittances (2.5%, 3.5%, 5.0%).

## 3. Results and discussion

**Fig 3:**
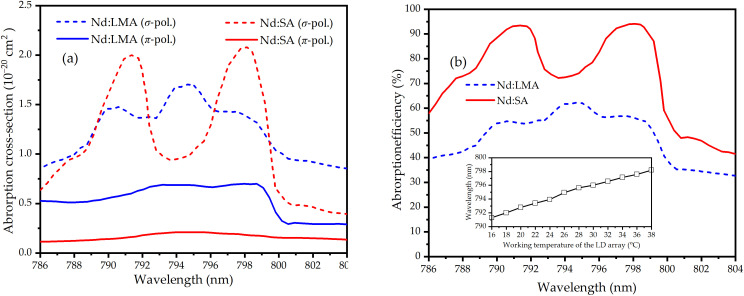
(a) Absorption (a) cross-sections and (b) efficiencies versus wavelength for Nd:LMA (6 mm, 1.0 at.%) and Nd:SA (10 mm, 2.0 at.%). Inset of Fig 3 (b): LD emission wavelength versus LD operating temperature.

Power balancing between Nd:LMA and Nd:SA crystals was achieved by sequential pump absorption: first in lower-gain Nd:LMA, then residual pump absorption in higher-gain Nd:SA. Absorption cross-sections (*σ*_*abs,i*_) across 786–804 nm were derived via Judd-Ofelt theory [63] using established parameters [56,61,64,65], as displayed in Fig 3(a). Their absorption efficiencies *η*_*abs*,*i*_ = 1-exp(−2*α*_*i*_*l*_*i*_) were derived as displayed in Fig 3(b). Here *i* = 1297, 1306 represents the two wavelengths of 1297 nm and 1306 nm, respectively, *α*_*i*_ = *σ*_*abs,i*_ *N*_*i*_, *N*_*i*_ is Nd^3+^ concentration in units of ion/cm^3^ (*N*_*i*_ are 2.95 × 10^19^ cm^-3^ and 6.73 × 10^19^ cm^-3^ for 1.0% doped (*N*_*ion*_) Nd:LMA and 2.0% doped Nd:SA, respectively), and *l*_*i*_ is the crystal length. The OPDW power output ratio adjustment was achieved through pump wavelength tuning or pump beam waist positioning control. Fig 3(b) inset shows the measured wavelength of the pump beam versus LD operating temperature. LD operating temperature variation (16°C → 38°C) shifted pump wavelength linearly across 791.1–798.2 nm, as displayed in the inset of Fig 3 (b). By adjusting the pump wavelength, dynamic control over the absorption power ratio between the two laser crystals can be achieved. This directly alters the output power at their respective emission wavelengths, enabling precise regulation of the output power ratio in the OPDW laser.

**Fig 4:**
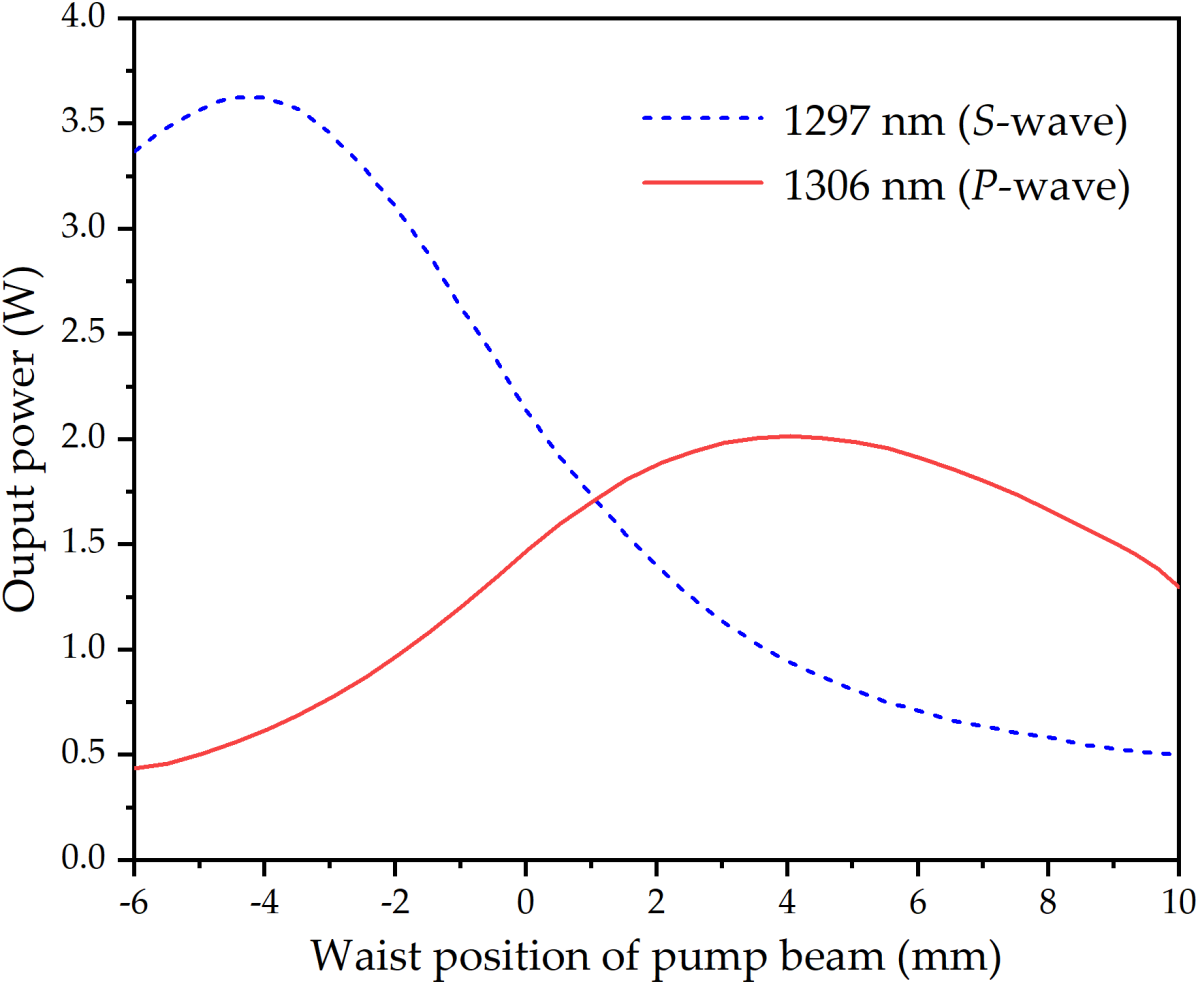
Calculated output powers of 1297 nm and 1306 nm versus pump beam waist location at LD operating temperature = 32 °C.

**Fig 5:**
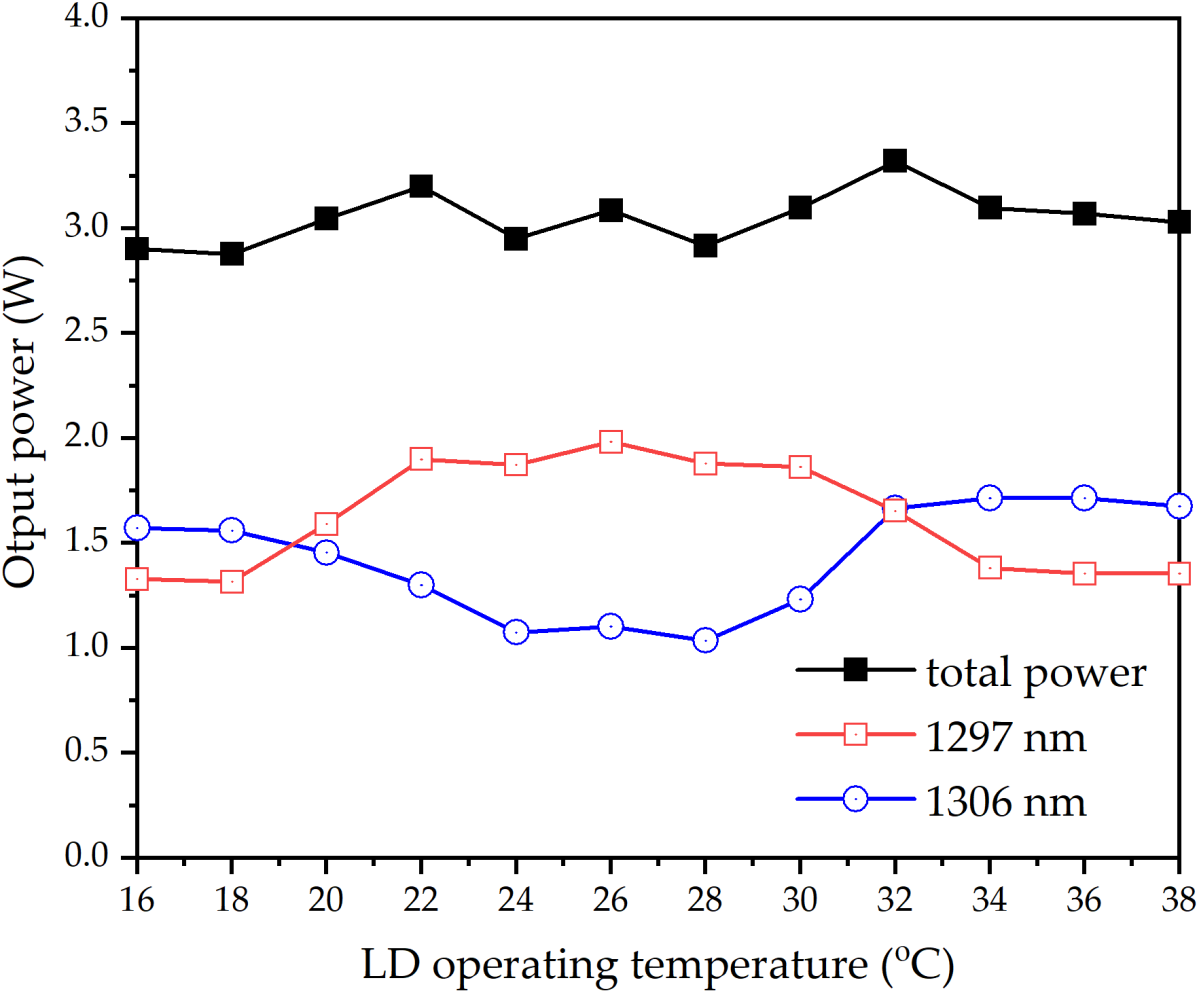
1297 nm and 1306 nm output power versus LD temperature (pump bean waist location: z = 1.0 mm and Pabs = 17.9 W).

**Fig 6:**
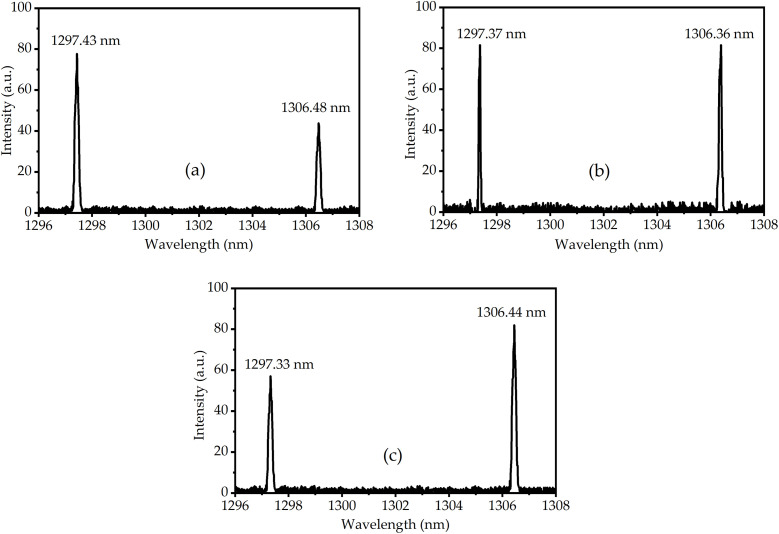
Laser spectra at 1297 and 1306 nm for three operating temperatures (a) at 26 °C, (b) 32 °C and (c) 38 °C.

End-pumped four-level solid-state laser systems have CW output power (*P*_*out,i*_) given by [66]:


$$P_{out,i}=\frac{1}{2}A_{i}T_{i}\frac{{hv}_{i}}{\sigma_{i}}\left(\frac{{2\sigma_{i}N_{i}W_{i}l}_{i}}{L_{i}}-W_{i}-\frac{1}{\tau_{i}}\right),$$ (1)

where *A*_*i*_ is the beam waist area at laser wavelengths, which was affected by the gain medium thermal lensing. The thermal focal lengths of the gain media were calculated using Ref. [67]. *T*_*i*_ is the output coupler transmittance, *hv*_*i*_ is the laser photon energy, σ_*i*_ is the emission cross-section, *τ*_*i*_ is the radiative lifetime, *L*_*i*_ is the round-trip loss, W is the pump parameter and can be written as [66]


$$W_{i}=\frac{\eta_{i}p_{i}\alpha_{i}}{N_{i}{hv}_{p}l_{i}}\int \frac{e^{-\alpha_{i}z}}{n_{i}\pi \omega_{p0}^{2}\left\{1+{\left[\frac{M^{2}\lambda_{p}}{n_{i}\pi \omega_{p0}^{2}}\left(z-z_{0}\right)\right]}^{2}\right\}}dz,$$ (2)

where *η*_*i*_ is the quantum efficiency, *p*_*i*_ is a pump power at the Nd:LMA input face, *p*_1297_ = *P* and *p*_1306 _=* *P** – *P* exp(–2*α*_1297_ *l*_1297_) when the pump power is **P.* ω*_*p*0_ is the pump waist radius, *hv*_*p*_ is the pump photon energy, M^2^ is the pump beam quality factor, *λ*_*p*_ is the wavelength of the pump beam, *n*_*i*_ is the refractive index of laser crystal. Z_0_ is the waist location of the pump beam, and Z_0_ = 0 is defined at the Nd:LMA and Nd:SA interface. With Eqs. (1) and (2) and the relevant experimental parameters: *T*_*i*_* *= 0.035, *ν*_1297 nm_* *= 2.31 × 10^14^ Hz, *ν*_1297_* *= 2.29 × 10^14^ Hz, σ_1297_* *= 0.55 × 10^−20^ cm^2^, σ_1306 _= 1.13 × 10^−20^ cm^2^, *L* = 0*.*05, σ_1297_* *= 401 μs [61], σ_1306 _= 436 μs [56], *P* = 18.7 W, *ω*_p0_ = 190 μm, M^2^ = 45, *λ*_*p*_ = 796*.*56 nm, *n*_1297 _= 2*.*21, *n*_1306_* *= 2*.*13, σ* *= 0.61. At LD operating temperature = 32 °C, ν_p_ = 3.77 × 10^14^ Hz, α_1297 _= 0.50 cm^-1^, α_1306 _= 1.2 cm^-1^, dependence of 1297 nm and 1306 nm output powers on pump beam waist position appears in Fig 4. It can be seen in Fig 4 that balanced power output was achieved at *z* = 1.0 mm (LD operating temperature: 32°C). Controlled lateral displacement of the pump waist from this position enabled precise adjustment of the OPDW laser power ratio. With pump beam waist fixed at *z* = 1.0 mm, Fig 5 shows the temperature-dependent output powers of 1297 nm and 1306 nm. Below 20°C or above 32°C, 1306 nm emission consistently surpassed 1297 nm due to enhanced excitation efficiency in Nd:SA crystals. Conversely, within 20–32°C, the power relationship reversed. It can be seen that total output power peaked at 32°C, declining monotonically with temperature deviation. This maximum corresponds to optimal excitation conditions for both crystals. Output spectra at representative temperatures (26°C, 32°C, 38°C) appear in Fig 6.

**Fig 7:**
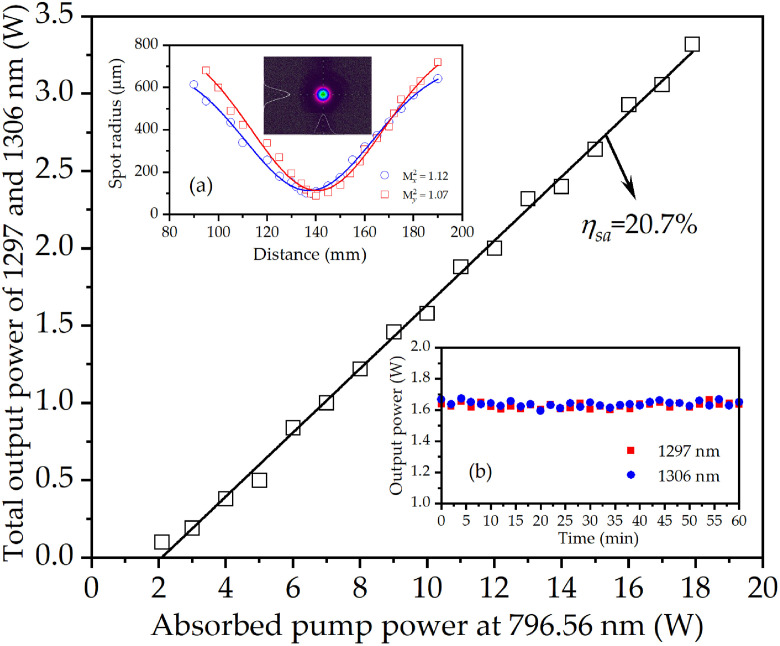
Total balanced power output of the 1297 nm and 1306 nm versus absorbed pump power at 796.56 nm. Inset **(a)**: output laser beam radii and shape. Inset **(b)**: power stabilities of the 1297 nm and 1306 nm.

Fig 7 shows the absorbed pump power versus total balanced power at 1297 nm and 1306 nm. The maximum total power output was 3.32 W at 17.9 W (= 56.5% × 18.7 W + 43.5% × 18.7 W × 90.2%) absorption. The laser system achieved 20.7% slope efficiency and 18.5% optical-to-optical conversion efficiency. At the maximum lasing output, the measured beam quality factors (M^2^) were 1.12 (*x*-axis) and 1.07 (*y*-axis), respectively. The inset (a) of Fig 7 shows the output laser beam radii and shape. Stability testing employed a power meter to record dual-wavelength outputs. At maximum power, RMS fluctuations over one hour measured 2.23% (1297 nm) and 2.31% (1306 nm), as shown in Fig 7(b) inset. Compared with the previous OPDWs based on physical bonding or diffusion bonding composite crystals [51,52,68], this study provides a simpler and more efficient system by using two independent crystals as gain medium. Furthermore, by optimizing the pump beam waist position within the gain medium, optimal excitation of both crystals is achieved when the output powers of the orthogonally polarized components reach a balanced state.

## 4. Conclusion

A compact and efficient OPDW laser is presented based on two independent crystals with perpendicular optical axes. Through gain controlling with tunable pump beam waist position or variable pump wavelength, the power ratio between two polarizations could be flexibly changed. A CW OPDW Nd:LMA/Nd:SA laser at 1297 nm and 1306 nm on the ^4^F_3/2_ → ^4^I_13/2_ transition was experimentally realized using an in-band LD pumping with tunable peak wavelength from 791.1 to 798.2 nm. The LD working temperature and pump beam waist position were optimized to achieve high efficiency and balanced output power in the OPDW laser. The maximum total output power of the OPDW laser at 1297 nm and 1306 nm reached 3.32 W, with a power ratio of 1:1. The maximum total slope efficiency and total optical-to-optical conversion efficiency with respect to the absorbed pump power at 796.56 nm were 20.7% and 18.5%, respectively. The structure of the OPDW laser is simple, and the stability and repeatability of the laser output are high. Compared with previously reported solid-state OPDWs requiring dual pump sources and complex cavities, this novel configuration offers significant advantages in compactness and cost. Additionally, it eliminates gain competition between dual transitions in a single gain medium. Further, through a carefully engineered traveling-wave resonator, single-longitudinal-mode operation for both polarizations can be achieved, paving the way for applications in precision interferometry and quantum metrology. We believe that the same technique presented in this paper can be applied to other composite crystals to achieve the OPDW lasers of other bands with adjustable output power ratio.
